# TECHNOLOGY FOR HEALTHCARE WORKFORCE FOR OLDER ADULTS: CHATGPT-ASSISTED QUALITATIVE ANALYSIS

**DOI:** 10.1093/geroni/igae098.4274

**Published:** 2024-12-31

**Authors:** Soojeong Han, Gregory Alexander

**Affiliations:** Columbia University School of Nursing, New York, New York, United States; Columbia University, New York, New York, United States

## Abstract

Rigorous guidance on ChatGPT use in qualitative research is not available. Here we examine the quality of ChatGPT versus human analysis. The International Summit on Innovation and Technology in Care of Older People (IS-ITCOP) was held on June 6-7, 2024 in New York. 47 experts in care of older adults attended IS-ITCOP. Attendees participated in structured roundtable discussions about long-term care workforce readiness to use technology in care of older adults. Each roundtable discussion included a lead facilitator and support staff who recorded discussions lasting approximately 2 hours. We used 10 roundtable discussions in our analysis. We followed the methodology established by Goyanes, Lopezosa, and Jordá(2024) for ChatGPT. Our human analysis was based on Braun and Clarke’s(2006) approach. Emerging themes included data privacy and security, informed consent, balancing autonomy and safety, role of the informatics workforce, technological advancements and ethical use, and policy and regulation. Compared to human analysis, 14 out of 79 sub-themes (18%) were missing in ChatGPT analysis. Findings suggest that ChatGPT has several strengths. ChatGPT completed the qualitative analysis in a few minutes, while humans took several days. ChatGPT provided clear definitions and explanations of words and concepts. It efficiently calculated convergence and divergence rates for each theme. ChatGPT also has weak points. ChatGPT cannot create a hierarchical coding scheme. It may not be able to capture all prompts from discussions. ChatGPT’s answers were derived from its training data, which may not be current. Also, ChatGPT appeared to miss some nuances or keywords, such as EHR.

